# Going viral? Linking the etiology of human prostate cancer to the *PCA3* long noncoding RNA and oncogenic viruses

**DOI:** 10.15252/emmm.201708072

**Published:** 2017-07-27

**Authors:** Andre A Teixeira, Serena Marchiò, Emmanuel Dias‐Neto, Diana N Nunes, Israel T da Silva, Bryce Chackerian, Marc Barry, Richard C Lauer, Ricardo J Giordano, Richard L Sidman, Cosette M Wheeler, Webster K Cavenee, Renata Pasqualini, Wadih Arap

**Affiliations:** ^1^ University of New Mexico Comprehensive Cancer Center Albuquerque NM USA; ^2^ Division of Molecular Medicine Department of Internal Medicine University of New Mexico School of Medicine Albuquerque NM USA; ^3^ Department of Biochemistry Chemistry Institute University of São Paulo São Paulo Brazil; ^4^ Department of Oncology University of Torino School of Medicine Torino TO Italy; ^5^ Candiolo Cancer Institute‐Fondazione del Piemonte per l'Oncologia (FPO) Istituto di Ricovero e Cura a Carattere Scientifico (IRCCS) Candiolo TO Italy; ^6^ Laboratory of Medical Genomics A.C.Camargo Cancer Center São Paulo Brazil; ^7^ Laboratory of Neurosciences Institute of Psychiatry University of São Paulo São Paulo Brazil; ^8^ Laboratory of Computational Biology A.C.Camargo Cancer Center São Paulo Brazil; ^9^ Department of Molecular Genetics and Microbiology University of New Mexico Albuquerque NM USA; ^10^ Department of Pathology University of New Mexico School of Medicine Albuquerque NM USA; ^11^ Division of Hematology/Oncology Department of Internal Medicine University of New Mexico School of Medicine Albuquerque NM USA; ^12^ Department of Neurology Harvard Medical School Boston MA USA; ^13^ Ludwig Institute for Cancer Research University of California‐San Diego La Jolla CA USA

**Keywords:** Cancer, Microbiology, Virology & Host Pathogen Interaction, Urogenital System

## Abstract

The hypothesis is discussed that prostate cancer marker lncRNA *PCA3* was introduced into the human genome by an oncogenic virus, and that viral infection‐related mechanisms might underlie its overexpression and prostate cancer initiation and/or progression.

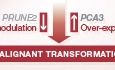

Prostate cancer, the most frequent type of cancer in men, is surpassed only by lung cancer in causing cancer‐related death. Despite major progress in defining the mutational landscape of this tumor, its etiology remains obscure. The intronic long‐noncoding RNA (lncRNA) *PCA3* is a specific marker of prostate cancer that acts as a trans‐dominant negative oncogene to down‐regulate the tumor suppressor gene *PRUNE2*. The unusual genomic organization and sequence of *PCA3* leads us to hypothesize that it was introduced into the human genome by an as‐yet undefined oncogenic virus. We further suggest viral infection‐related mechanisms as functional in *PCA3* overexpression and involved in prostate cancer initiation and/or progression. This is supported by known links between viral infections and prostate cancer, and the lack of this tumor in other mammals despite its high incidence in humans. Finally, we suggest that genomic sequence‐based approaches might help us uncover the potential role of viruses in prostate cancer etiology.

## Introduction: The curious case of *PCA3* in prostate cancer

The intronic lncRNA *Prostate Cancer Antigen 3* (*PCA3*) is a highly specific biomarker: While only present at low levels in normal or benign hyperplastic prostate, its expression is increased up to 100‐fold in about 95% of prostatic carcinomas (Schalken *et al*, 2003). Despite this strong correlation, the biological function of *PCA3* remained elusive for almost two decades after its discovery (Bussemakers *et al*, 1999), until we established its role as a trans‐dominant negative oncogene that downregulates a protein‐coding tumor suppressor gene, *prune homolog 2* (*PRUNE2*) (Salameh *et al*, 2015). In experimental models of human prostate cancer, *PCA3* overexpression as well as *PRUNE2* silencing accelerates the growth of tumor xenografts *in vivo*, while increasing cell proliferation, adhesion, and migration *in vitro* (Salameh *et al*, 2015). In prostate cancer patients, decreased levels of *PRUNE2* are indicative of poor prognosis, as this is associated with metastatic disease (National Center for Biotechnology Information, Gene Expression Omnibus, NCBI GEO, profile 34856174; *n* = 171) and a high Gleason score (The Cancer Genome Atlas, TCGA; *n* = 497). Surprisingly, data retrieved from large‐scale databases of human tissue samples (TCGA; and The Atlas of noncoding RNAs in Cancer, TANRIC) show homogeneous expression of *PCA3* only in prostate cancer, but not in other tumor types, including those in which *PRUNE2* loss‐of‐function events have been reported, that is, Merkel cell carcinoma, neuroblastoma, glioblastoma, and parathyroid carcinoma.

From an evolutionary standpoint, *PCA3* is a relatively new gene: All four exons and the upstream (putative promoter‐containing) sequence are conserved among primates, but only exon 4 shares conservation with the rabbit (68% identity) and mouse (52% identity) orthologs. Prostate cancer occurrence is even more stringent than *PCA3* conservation: Despite its high incidence in humans, it is rare or absent in other mammals (De Marzo *et al*, 1999), including nonhuman primates kept in captivity (ruling out the possibility that a shorter lifespan might account for the difference). Dogs are the only known exception, but even here the incidence is much lower than in humans. Moreover, while the vast majority of prostate cancers in men are androgen‐dependent adenocarcinomas, dogs more often present with androgen‐independent ductal carcinomas. A simple link between human diet and predisposition to prostate cancer (De Marzo *et al*, 1999) also does not satisfactorily explain the paradoxical (non)‐diffusion of this tumor. The intriguing association between *PCA3* and prostatic carcinoma in human males is at odds with the established concept that oncogenes are shared among different tumor types and/or animal species. How might this tissue‐ and species‐specificity be linked to the currently unknown triggering event(s) in prostate cancer?

## A connection between oncoviruses, long noncoding RNAs, and cancer: coevolution to optimize fitness

Tumor‐related lncRNA transcripts are often encoded and/or regulated by viruses. Indeed, all known oncoviruses, including hepatitis B and C viruses (HBV and HCV, respectively), Epstein–Barr virus (EBV), human papillomavirus (HPV), and Kaposi's sarcoma‐associated herpesvirus (KSHV), produce their own lncRNAs and/or induce the transcription of cellular lncRNAs, contributing to cancer predisposition (Li *et al*, 2016). This “virus‐lncRNA‐cancer” *leitmotif* has been validated in different malignant settings including HBV‐*Highly Upregulated in Liver Cancer* (*HULC*) in hepatocellular carcinoma, EBV‐*Small Nucleolar RNA Host Gene 8* (*SNHG8*) in gastric cancer, and HPV‐*HOX transcript antisense intergenic RNA* (*HOTAIR*) in cervical cancer. The connection is not limited to ongoing infections: Ancient viruses and their animal hosts have actively exchanged portions of genetic material throughout evolution (Krupovic & Koonin, 2017). This phenomenon is related to the need of viruses to optimize their replication by exploiting the eukaryotic cell machinery. In addition, random viral insertions can lead to the formation of virus‐host chimeric functional units (e.g., lncRNAs) that are selected for their capacity to favor host functions in physiological and/or pathological conditions (e.g., cancer, inflammation, immunity). A consequence of this coevolution, the conservation between viral sequences and lncRNAs in at least mammals and birds, is beginning to be unraveled with regard to genomic structure and regulation. An interesting example in the poultry industry is the integration of several related avian leukosis viruses with miRNAs and lncRNAs to cause tumors, decreased fertility, and premature death (Lan *et al*, 2017).

## Is *PCA3* an oncovirus‐derived gene?

Several attributes of *PCA3* suggest that this lncRNA may be derived from and/or regulated by viruses. First, the *PCA3* promoter and exon 1 are included in a partially conserved long interspersed nuclear element type 2 (LINE‐2) repeat, which is an ancient virus‐derived retrotransposon. This is similar to the HBV‐regulated lncRNA *HULC*, whose promoter and first exon are embedded in a long‐terminal repeat (LTR) retrotransposon‐like sequence (Kapusta *et al*, 2013). Second, as would be expected if the PCA3 promoter were derived from a virus, it does not contain canonical transcription factor binding sites (e.g., TATA box) and shows no sequence conservation with any annotated human promoter. Third, *PCA3* is located on the opposite DNA strand to intron 6 of the *PRUNE2* gene. This configuration is reminiscent of the bidirectional transcription of the EBV latency origin of replication, with leftward transcripts being mainly noncoding and regulatory, and rightward transcripts containing open reading frames (Cao *et al*, 2015). Fourth, *PRUNE2* downregulation by *PCA3* occurs through adenosine deaminase acting on RNA (ADAR)‐mediated editing, a post‐transcriptional mechanism largely employed in the cellular response to viruses such as polyomavirus and EBV (Cao *et al*, 2015), and upregulated in EBV‐infected lymphocytes (http://www.gtexportal.org/home/gene/ADAR).

## A tale of sex, viral transmission, and human prostate cancer

Human prostate cancer has been associated with several viruses. Digital expression studies have shown that the human endogenous retrovirus K (HERV‐H), a remnant of ancient infections fixed in the ancestral human genome 30–70 millions of years ago, is overexpressed in human stomach, colon and prostate cancers. The activation of an analogous retrovirus, HERV‐K, induces the production of corresponding antigens and immune responses (Reis *et al*, 2013) that correlate with prostate cancer progression. A few additional indications of a direct viral etiology also exist. The BK polyomavirus (BKV) seems to be a cofactor in early stages of prostate cancer (Das *et al*, 2008), and its correlation with malignant progression has been proposed. Overall, a 19% prevalence of polyomaviruses such as BKV, JC virus (JCV), and SV40 has been reported in prostate cancer cases, suggesting that the subclinical persistent infections that are frequent in the human population might contribute to the onset and/or progression of this malignancy. Positive association with other oncoviruses such as HPV has also been established. In general, an increased relative risk estimate for prostate cancer has been associated with sexually transmitted diseases (e.g., gonorrhea and syphilis) (Dennis & Dawson, 2002), supporting the possibility of infectious components in tumor development. Despite their limitations, these data are encouraging: The challenge of finding a direct link between viral infection and cancer is common to a number of pathologies for which standard causation rules, for example, Koch's postulates and Hill's criteria of causality (reviewed in Moore & Chang, 2014), do not apply. Heterogeneous etiologies have been described for other virus‐linked cancers, for example, Merkel cell carcinoma, where the presence of polyomavirus alone is not sufficient for tumor development as well as squamous cell carcinomas of the genital and head‐and‐neck regions, in which HPV infection appears to be a necessary yet insufficient cause (zur Hausen, 2002).

## Conclusion: How could this hypothesis be tested?

To actually demonstrate the connection between viral infection, lncRNAs, and prostate cancer, the characterization of the variety of viruses present in tumor cells will be needed. Digital transcript subtraction has been successfully applied to uncover the association between polyomavirus and human Merkel cell carcinoma (Feng *et al*, 2008) and could also be employed to hunt for new oncoviruses in prostate cancer. To cover the full spectrum of virus‐related mechanisms, a properly designed variant of this *in silico* strategy should be designed to include noncoding RNAs. Such next‐generation approaches would rule out potential contaminations, as in the unfortunate precedent of xenotropic murine leukemia‐related virus (XMRV), a putative retrovirus involved in prostate cancer later demonstrated to be a laboratory artifact (Paprotka *et al*, 2011). Also, a systematic analysis of virus‐derived genomic sequences in a large cohort of prostate cancer patients would elucidate the structural connections between lncRNAs and ancient infections, as a preliminary step toward a comprehensive functional characterization of the corresponding mechanisms. Above all, this analysis should allow low stringency homology searches, to include poorly conserved sequences (such as the LINE‐2 repeat in which *PCA3* is embedded) derived from millions of years of evolutionary pressure, and thereby barely recognizable.

**Figure 1:**
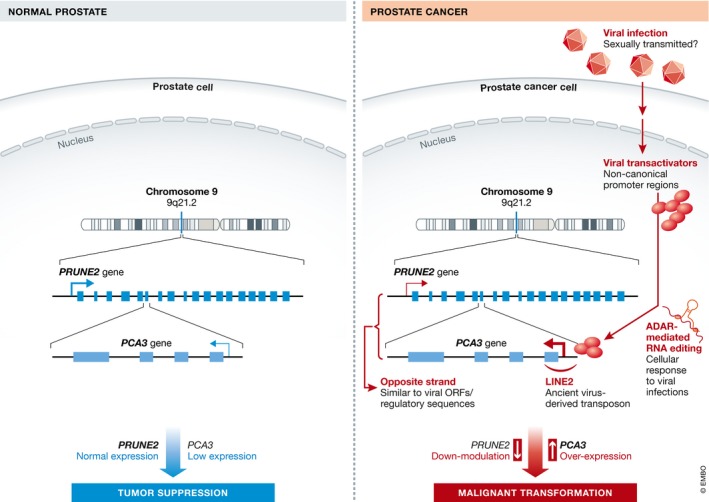
Schematic view of the proposed viral etiology of prostate cancer In the normal prostate, basal expression of *PRUNE2* correlates with low *PCA3* levels. In prostate cancer, a putative oncovirus could activate *PCA3* expression through a direct transactivation of a cryptic, ancient virus‐derived promoter, resulting in downmodulation of *PRUNE2*, and leading to dysregulated cell proliferation and acquisition of malignant attributes.

In summary, we hypothesize that the prostate‐specific lncRNA *PCA3* has been introduced into the human genome by an ancient virus, and that it is regulated by virus‐specific mechanisms (Fig 1). The identification of cellular and/or viral regulatory processes that are specifically engaged in prostate pathophysiology will be pivotal for understanding the contribution of *PCA3* and other noncoding RNA transcripts in the onset and progression of prostate cancer, as well as the peculiar restriction of this tumor to humans.

## Conflict of interest

RP and WA have a patent 15/500,686 licensed to Mbrace Therapeutics on the subject of the present manuscript. All other authors declare that they have no conflict of interest.
